# Characteristics of People Seeking Prescribed Cannabinoids for the Treatment of Chronic Pain: Evidence From Project Twenty 21

**DOI:** 10.3389/fpain.2022.891498

**Published:** 2022-06-14

**Authors:** Anne Katrin Schlag, Michael Lynskey, Alan Fayaz, Alkyoni Athanasiou-Fragkouli, Brigitta Brandner, Barbara Haja, Elizabeth Iveson, David J. Nutt

**Affiliations:** ^1^Drug Science, London, United Kingdom; ^2^Department of Psychiatry, Imperial College London, London, United Kingdom; ^3^Anaesthesia and Pain Medicine, University College London Hospital National Health Service (NHS) Foundation Trust, University College London, London, United Kingdom; ^4^Stroke and Neurorehabilitation, Nuffield Health, London, United Kingdom

**Keywords:** chronic pain, cannabinoids, cannabis based medicinal products (CBMPs), real world evidence (RWE), Project Twenty 21 (T21)

## Abstract

**Background:**

Prescribed cannabinoids are now legal in the UK and increasingly being used for a variety of conditions, with one of the most frequent conditions being chronic pain. This paper describes the characteristics of individuals seeking prescribed cannabinoids for the treatment of chronic pain in Project Twenty 21, a UK based real world data registry of prescribed cannabis patients.

**Method:**

By 1st November 2021 data were available for 1,782 people who had sought treatment with medical cannabis as part of Project Twenty 21. The most common diagnosis among this cohort was chronic pain with 949 (53.5%) of the cohort reporting a primary condition related to chronic pain. Medical and self-report data on the characteristics of these patients, their health status and type/s of cannabinoid/s prescribed are summarized in this report.

**Results:**

Of the 949 people reporting chronic pain as a primary condition 54.7% were male and their average age was 42.0 years (range = 18–84). Patients reported a low quality of life and high levels of comorbidity: people reported an average of 4.6 comorbid conditions with the most common comorbid conditions including anxiety, depression, insomnia and stress. A range of cannabinoid products were prescribed with the most common products being classified as high THC flower (48.5%). The majority of patients also reported using at least one other prescribed medication (68.7%).

**Conclusions:**

Consistent with findings in other national and international databases, chronic pain was the most common primary condition in this real world study of prescribed cannabinoids. There was considerable variation in the types of chronic pain, comorbid pathology and in the characteristics of products being prescribed to treat these conditions. Together, this evidence supports the utility of real world evidence, as opposed to clinical trial approaches to studying the potential benefits of prescribed cannabinoids in treating chronic pain.

## Introduction

Chronic pain is a pervasive, and potentially disabling condition. In the UK it is estimated to affect between one third and one half of the adult population, contributing to major social and economic costs (1). Pain, by definition, is an aversive sensory and emotional experience, but persistent symptoms are likely to be associated with limitations in function (2, 3), altered mood (4), poor sleep (5, 6) and marked reduction in perceived quality of life (2, 7). In view of the systemic impact pain symptoms may have, a holistic, patient-centered management approach is often favored—addressing not just the biological, but also the psychological and social needs of those affected. Biological treatments may include pharmaceuticals, interventional and manual therapies. However, despite considerable advances in our understanding of pain mechanisms over the past half a century, there remains a lack of effective pharmacological treatments for chronic pain symptoms.

### Medical Cannabis and Pain

Cannabis-based medical products (CBMPs) are approved in over 40 countries and prescribed for a broad variety of conditions. In the UK, CBMPs became legal in November 2018, nevertheless prescribing here remains very limited (8). The National Institute for Care and Excellence (NICE) does not recommend the prescription of CBMPs for chronic pain. Reasons for this are varied and complex, including considerable medicolegal and bureaucratic hurdles (9). But also there is concern by the medical profession that the randomized control trial (RCT) evidence base for medical cannabis in relation to pain is limited: ranging from weakly positive (10–13) to inconclusive or negative (14–17).

However, existing studies often do not adequately include or assess patient reported outcomes (PROs). Rather RCTs tend to use pain intensity ratings as the primary outcome in their trials, potentially underestimating the impact of treatments such as CBMPs, that do not just affect pain *per se*, but also the overall quality of life of the patient (18). In a recent multi-criteria decision analysis by Nutt et al. that included patient user representatives the perceived benefit-safety profiles for cannabinoids were higher than for other commonly used medications for chronic neuropathic pain largely because they contribute more to quality of life and have a more favorable side-effect profile (19). These findings reflect the shortcomings of trial data in pain treatments where primary outcomes are centered purely around reductions in pain intensity.

### Current Real World Evidence

Real World Evidence (RWE) consistently shows that various pain conditions are by far the most common conditions for which cannabis is prescribed (20). Currently, up to 90% of patients in state-level medical cannabis registries list chronic pain as their qualifying condition for the medical program (21). Emerging observational data of UK based registries corroborate these findings (22, 23). Additionally, an increasing number of observational studies highlight that CBMPs may be used as an alternative treatment by intermittent or chronic opioid users to mitigate their pain (24).

The widespread availability of prescribed cannabinoids in many jurisdictions, limitations of RCTs for the study of effects of complex and varied medications such as cannabinoids and the range of conditions for which they are prescribed, highlight the potential of real world research for studying the potential benefits and safety of these drugs.

### Project Twenty 21

Launched in August 2020, Project Twenty 21 (T21) is a multi-center, prospective, observational patient registry of RWD for patients seeking treatment with prescribed cannabinoids. A previous paper offers a detailed description of the methodology of our study (23). The over-arching goal of this project is to collect prospective data from substantial numbers of people who receive CBMPs for a variety of conditions, in order to contribute to both the scientific literature and regulatory aspects on the safety and effectiveness of these products in real-world settings. The benefits of widening the scientific evidence base on medical cannabis to also include observational data, in addition to RCTs, are potentially substantial.

Cannabis is a complex medicine, and the broad variety of compounds in different ratios, means assessing the evidence through RCTs would take many years to complete. In contrast, large numbers of patients have been self-medicating with illicit cannabis-based products for medicinal use (25) and the international database evidence suggest this new class of drugs offers a significant advance in treatment for many in whom current medicines are either ineffective or poorly tolerated (26).

In this study we present data on patient characteristics and prescribed products for individuals within the cohort who reported a primary diagnosis of chronic pain.

## Methods

### Recruitment Strategy and Consent

At the time of writing, UK regulations for receipt of prescribed cannabis stipulated that an individual must have an established diagnosis and evidence of failure of at least two treatment approaches before being eligible to legally receive prescribed CBMPs. Therefore, individuals receiving a prescription would principally need an established history of not just chronic, but also treatment resistant symptoms. There were no inclusion or exclusion criteria specifically for participation in the registry; all patients receiving a prescription for CBMPs for the indications listed below were eligible to join the registry. Patients were able to self-refer to a prescribing physician at a clinic of their choice. However, they were required to present all necessary documentation confirming their diagnosis from their general practitioner or other clinician, including:

- Evidence of diagnosis meeting one of seven clinical indications (i) chronic pain, (ii) post-traumatic stress disorder (PTSD), (iii) anxiety, (iv) multiple sclerosis, (v) Tourette's syndrome and (vi) substance use disorder or (VII) epilepsy.
- Past medical history and comorbidities.
- Current medications.

Eligible patients are entered into the registry and followed for at least 2 years for data collection purposes at the same intervals used in standard of care (every 3 months). Decisions about the suitability of CBMPs for a specific individual are entirely the responsibility of the treating physician. All individuals provide signed, informed consent.

### Cannabis Based Medical Products

The Twenty 21 formulary contains 22 products including both oils and flowers and a range of CBD: THC ratios. It is also possible for clinicians to prescribe other cannabis products. Based on the known properties of these products they were classified into: high CBD oils (low or no THC); balanced CBD: THC oils; high THC (low or minimal CBD oils); high CBD flower (low or no THC); balanced CBD: THC flower; high THC (low or minimal CBD) flower.

#### Prescribed Medications

Information was collected on all prescribed medications that the individual was currently using. We classified these medications as: paracetamol, opioids-weak, opioids-strong, analgesic-other, anti-neuropathic-conventional, anti-neuropathic-other, non-steroidal anti-inflammatory drugs (NSAIDs), NMDA receptor antagonists (ketamine), cannabis and other.

### Measures

As part of their clinical assessment, in addition to providing their medical history and past and current treatments, patients completed a number of structured assessments of symptomatology, based on standardized and well-validated self-report questionnaires (23). The measures analyzed in this report included:

#### Chronic Pain

Patients who reported their primary condition as chronic pain were required to complete the brief pain inventory (27). This scale includes 4 items assessing pain intensity and a further seven items assessing the extent to which pain interferes in the individual's daily activities.

#### Depression

Depression was assessed using the PHQ-9 questionnaire (28), a nine-item scale which assesses the frequency of depressive symptomatology during the preceding 2 weeks on a four-point scale (0 = not at all; 1 = several days; 2 = over half the days; 3 = nearly every day) (29). These items can be summed to form an overall measure of depressive symptomatology.

#### Quality of Life

Quality of life was assessed using the EQ-5D-5L (30), which contains five items, each assessed on a five-point scale, assessing mobility, self-care, capacity to complete usual activities, pain and anxiety/depression. The EQ-5D−5L also contains a visual analog scale (0–100) assessing current health with 0 meaning the worse health they can imagine while 100 means the best health they can imagine.

#### Sleep Quality

Quality of sleep was assessed using four items derived from the Pittsburgh Sleep Quality Index (31), each of which was assessed on a five-point scale: (a) How much sleep patterns were interfering with daily activities (not at all, a little, somewhat, much, very much); (b) Difficulties falling asleep, (c) difficulties staying asleep and; (d) waking up too early were each assessed using the scale: none, mild, moderate, severe, very severe.

#### Statistical Analysis

In this report we use descriptive statistics to report proportions (%) of people reporting specific conditions and means to summarize ratings on standardized scales.

## Results

By 1st November 2021, data were available for a total of 1,782 individuals seeking treatment with medicinal cannabinoids. Chronic pain was the most common primary condition among this sample and was reported by 949 individuals (53.5% of the sample), followed by anxiety disorders (33.4%) while smaller minorities reported post-traumatic stress disorder (6.9%), multiple sclerosis (3.0%), epilepsy (0.9%), substance use disorders (0.7%) and Tourette's syndrome (0.6%).

### Sample Characteristics for Patients Reporting Chronic Pain

The data showed a higher proportion of male patients seeking medical cannabis for chronic pain (57.4%), reflecting the general preponderance toward male patients across the whole data set (all indications; male 63.2%). The mean age of the chronic pain patients was 42.0 (SD = 12.7; range = 18–84) years.

In addition to chronic pain, 91.7% of those with a primary condition of chronic pain reported experiencing at least one comorbid secondary condition. Among those reporting secondary conditions: 34.2% reported 1–2 conditions; 31.5% reported 3–5 conditions and 34.4% reported 6 or more secondary conditions. The most commonly reported non-pain secondary conditions were: anxiety (41.9%), depression (29.9%), stress (23.7%) and insomnia (23.1%).

### Prior Experience With Cannabis

Only 14.4% of those with chronic pain reported that they had not previously used cannabis while nearly two thirds (61.6%) reported that they were currently using cannabis to treat their pain; 86.3% of these (52.7% of all pain patients) reported that they were using it daily.

### Self-Reported Health

#### Brief Pain Inventory Scores at Entry to Treatment

##### Pain Intensity

Pain intensity was assessed using the mean of the four intensity items contained in the Brief Pain Inventory (27). The sample reported high levels of pain with the mean pain intensity rating being 5.9 (SD = 1.6). Table 1 categorizes pain intensity using the cut-offs recommended by Serlin et al. (32): 45.4% of the sample were classified as experiencing severe pain.

**Table 1:** Distribution and classification of pain intensity and pain interference scores from the Brief Pain Index (*N* = 949).

	**Classification**	**Score**	**Percentage (%)**
Pain intensity	Mild	1–4	14.9
	Moderate	5–6	39.7
	Severe	7–10	45.4
Pain interference	Mild	<2	2.2
	Moderate	2–6	25.3
	Severe	>6	72.5

*Classification of pain intensity based on Serlin et al. (32). Classification of pain interference based on Shi et al. (33)*.

##### Pain Interference

The sample also reported high levels of pain interference (mean rating across the seven items was 7.0) (SD = 2.0) Categorizing reported pain interference using the cut-points recommended by Shi et al. (33) indicated that 82.1% of this were experiencing severe interference from chronic pain.

##### Depression

Table 2 shows a summary of the distribution of scores on the PHQ-9 assessment of depression, along with suggested diagnostic categories. 42.0% of the sample reported moderately severe (21.2%) or severe (20.8%) depression while only 11.2% of the sample reported no or minimal symptoms of depression.

**Table 2:** Distribution of PHQ-9 depression items and depression classifications (*n* = 898).

**PHQ-9 scores**	**Depression classification**	**% of sample**
0–4	None/minimal	11.2%
5–9	Mild	23.1%
10–14	Moderate	23.7%
15–19	Moderately severe	21.2%
20–27	Severe	20.8%

##### Sleep

All patients reported the quality of their sleep using four items derived from the Pittsburgh Sleep inventory: among those with a primary condition of chronic pain: 63.7% reported that their sleep patterns interfered with their daily activities much or very much, while 40.4% reported severe/very severe problems falling asleep, 43.7% experienced severe/very severe problems staying asleep and 35.9% reported severe/very severe problems waking up too early.

##### Quality of Life

Table 3 summarizes self-reported ratings across the five dimensions of the EQ-5D-5L. Each of these dimensions is assessed on a 5 point scale with one representing no or minimal problems while 5 represents extreme problems. Not surprisingly given this was a sample of people seeking treatment for chronic pain, a high percentage of the sample reported experiencing severe or extreme problems due to pain (55.5%). Additionally, relatively high percentages of people reported severe problems across the other four domains: 31.4% reported severe problems with mobility or being unable to walk; 12.0% reported severe problems with self-care or being unable to wash; 41.5% reported severe problems or being unable to complete usual activities and 17.8% reported severe or extreme problems with anxiety and depression. In contrast, between 5.3% (for usual activities) and 28.9% (for self-care) reported no problems on these individual dimensions.

**Table 3:** Mean scores on EQ-5D-5L dimensions among people with a primary diagnosis of chronic pain (*n* = 903).

**Extent of problems**	**Mobility**	**Self-care**	**Usual activities**	**Pain**	**Anxiety/depression**
None	12.6	30.3	5.3	0.7	21.5
Slight	21.7	29.7	15.6	8.7	31.7
Moderate	34.2	28.0	37.5	35.1	29.2
Severe	28.0	10.3	32.3	41.5	13.0
Unable to walk/wash/usual activities/ extreme	3.4	1.7	9.2	14.0	4.7

In addition, the EQ-5D-5L assesses general health using a 0–100 visual analog scale with 100 representing the best health imaginable. The mean score on this scale was 45.6 (SD = 19.6), representing relatively poor general health. In comparison, normative data for the UK household population reports a mean of 85.7 (out of 100) (34).

### Product Characteristics

53.8% of chronic pain patients received a prescription for a single CBMP, 35.6% received two products and 10.7% received three or more products. The types of products prescribed are summarized in Table 4: nearly half of all prescribed products were classified as high THC flower while nearly a quarter of all products were balanced oils. A further 10% of prescribed products were classified as high CBD oil while prescriptions for both high THC oil and high CBD flower were relatively uncommon.

**Table 4:** Characteristics of cannabis products prescribed to 949 people with chronic pain.

	**Oil**	**Flower**
High CBD (no/minimal THC)	10.3%	0.4%
Balanced CBD: THC	23.4%	14.1%
High THC (Low/minimal CBD)	3.3%	48.5%

### Other Prescribed Medicines Currently Being Used

The majority (68.7%) of participants reported that they were currently using at least one prescribed medication while many reported using multiple medications: 19.0% reported one medication, 17.1% reported two, 39.0% reported three to five and 24.9% reported using six or more prescribed medications. Among individuals using prescribed medications the mean number of current medications prescribed was 4.2 (range = 1–23 medications). The types of prescribed products being used is summarized in Table 5: conventional neuropathic agents were the most commonly prescribed medication while substantial minorities of the sample were in receipt of prescribed analgesics including weak (26.6%) and strong (14.1%) opioids. A large number of patients were receiving other prescribed medications, including those used to treat a range of chronic conditions including diabetes, hypertension and hypercholesterolaemia.

**Table 5:** Percentage of chronic pain patients using different types of prescribed medications (*N* = 949).

**Drug type**	**Example**	**Percentage of patients reporting use**
Paracetamol	Paracetamol	10.6%
Opioids—weak	Tramadol, Codeine, Dihydrocodeine	26.6%
Opioids-strong	Fentanyl, Oxycodone, Morphine, Methadone	14.1%
Analgesics—other	Diazepam, Baclofen	21.4%
Anti-neuropathic-conventional	Amitriptyline, Pregabalin, Conventional Gabapentin, Duloxetine	33.1%
Anti-neuropathic—other	Venlafaxine, Sertraline, Mirtazapine, Capsaicin	12.6%
NSAIDs	Ibuprofen, Naproxen, Diclofenac, Aspirin	19.2%
NMDA antagonists	Ketamine	0.3%
Cannabis	Noidecs, Satoline	2.4%
Other	Metformin, Cetirizine, Sumatriptan, Bisoprolol, Omeprazole	42.1%

## Discussion

Chronic pain is a common condition that is increasingly managed with CBMPs. Our data supports findings of other RWE databases (22), and international evidence that, with changes in the legal status and availability of these medications, they are increasingly being used to treat pain and other conditions.

In addition to chronic pain, the majority of our sample experienced at least one comorbid secondary condition, with over a third reporting 6 or more secondary conditions. Many of these secondary conditions were psychiatric conditions, such as anxiety and depression, which are also treated with CBMPs (20, 25). The high number of co-morbid conditions experienced also highlights the benefits of observational databases and RWE as many of these patients would have automatically been excluded from more formal RCTs.

Moreover, nearly half of our sample were classified as experiencing severe pain, as well as high levels of pain interference. Our data clearly show that our pain patients are very unwell, with low self-reported health and a low quality of life compared to the average population. Over half of the current sample experience severe or extreme problems due to pain. The low quality of life of patients, their high number of comorbidities and their wide age range shows that the myth of medical cannabis patients as young, recreational users looking for a legal source of cannabis can be firmly dispelled.

Quality of life is a key topic requiring further investigation in relation to medical cannabis and pain. It is one of the important outcome domains being measured in the evaluation of pain treatment effectiveness in general and has been suggested to be indicative of treatment success (35). An increasing literature highlights that medical cannabis can improve patients' quality of life (36, 37). In their survey study of Israeli medical cannabis patients with a diagnosis of chronic non-cancer pain, found that although pain intensities did not improve significantly during the study, quality of life did improve, and the rate of analgesic medication consumption decreased alongside with increasing rates of high dose THC and α-pinene consumption (36).

Our findings provide an initial indication of the range of CBMPs used by UK patients at present.

Further research needs to show if and how pain relief and side effect manifestation may vary across available cannabis product types. In order to be able to offer the best treatment plan, research is needed on the short and long term effectiveness and safety of individual cannabis products for specific types of chronic pain Whole plant cannabis products contain many other biologically active constituents, in addition to THC and CBD, such as terpenoids which may impact clinical outcomes (37). Understanding the exact composition of the CBMP may shed further light on its therapeutic value. As with other drugs, CBMPs need to be embedded in a multimodal treatment plan, with appropriate safeguarding support, and ongoing pharmacovigilance such as provided by Twenty 21.

In addition to CBMPs, the majority of participants reported that they were currently using at least one prescribed medication while many reported using multiple medications, including both weak and strong opioids. A growing body of academic research suggests that individuals are using cannabis as a substitute for prescription drugs, particularly, narcotics/opioids (38, 39). This is especially true if patients suffer from pain, anxiety and depression (40). As 5% of our sample report “opioid dependence” as secondary condition, future publications will analyse the impact of medical cannabis use on opioid sparring for our sample.

### Limitations and Future Research

This study has limitations like other observational studies and there is no control group. However, the main aim of our present study was to characterize in detail the pain patients receiving CBMPs through Project Twenty 21. The longer-term efficacy and safety of CBMPs on pain, as well as on other conditions, will be addressed in future publications.

## Conclusions

Our results confirm the complexity underlying the patient experience and the impact of chronic pain. The pharmacological management of chronic pain remains a major medical challenge, and it is important to include the clinical experience of patients and clinicians to inform treatment pathways and specific treatments. Our findings reflect results from other national and international databases, building up to a pattern of evidence, but the value of CBMPs for pain management remains controversial. In the UK, the relative lack of expert-based recommendations, clinical experience, education and patient support for these medicines still presents a challenge for patients and clinicians.

The long-term impact of CBMPs, for chronic pain as well as for other conditions, still needs to be fully understood. Longitudinal RWE studies, as reported here, can contribute to the scientific evidence base on CBMPs, which should be acknowledged by healthcare professionals and policy-makers, when formulating decisions about prescribing medical cannabis.

## Data Availability Statement

The raw data supporting the conclusions of this article will be made available by the authors, without undue reservation.

## Ethics Statement

Ethical review and approval was not required for the study on human participants in accordance with the local legislation and institutional requirements. The patients/participants provided their written informed consent to participate in this study.

## Author Contributions

ML and AKS developed the initial manuscript. AF wrote the sections on pain. ML, AA-F, BH, and AKS analyzed the data. EI, BB, and DJN provided clinical input. All authors revised the manuscript and agreed on the final version.

## Conflict of Interest

DJN is Chair of the charity Drug Science. ML is Chief Research Officer of Drug Science. AKS is Head of Research of Drug Science. AA-F is study co-ordinator of Project Twenty 21. AF, BB, and EI are members of the Drug Science Medical Cannabis Working Group. Drug Science receives an unrestricted educational grant from a consortium of medical cannabis companies to further its mission, that is the pursuit of an unbiased and scientific assessment of drugs regardless of their regulatory class. All Drug Science committee members, including the Chair, are unpaid by Drug Science for their effort and commitment to this organization. AKS is scientific advisor to the Primary Care Cannabis Network, and an executive member of the Cannabis Industry Council, both unpaid roles. None of the authors would benefit from the wider prescription of medical cannabis in any form. The remaining authors declare that the research was conducted in the absence of any commercial or financial relationships that could be construed as a potential conflict of interest.

## Publisher's Note

All claims expressed in this article are solely those of the authors and do not necessarily represent those of their affiliated organizations, or those of the publisher, the editors and the reviewers. Any product that may be evaluated in this article, or claim that may be made by its manufacturer, is not guaranteed or endorsed by the publisher.

## References

[1] Fayaz A Croft P Langford RM Donaldson LJ Jones GT. Prevalence of chronic pain in the UK: a systematic review and meta-analysis of population studies. BMJ Open. (2016) 6:e010364. 10.1136/bmjopen-2015-010364 27324708 PMC4932255

[2] Hadi MA McHugh GA Closs SJ. Impact of chronic pain on patients' quality of life: a comparative mixed-methods study. J Patient Exp. (2019) 6:133–41. 10.1177/2374373518786013 31218259 PMC6558939

[3] Vos T Abajobir A Abate KH Abbafati C Abbas KM Abd-Allah F. Global, regional, and national incidence, prevalence, and years lived with disability for 328 diseases and injuries for 195 countries, 1990-2016: a systematic analysis for the Global burden of disease study 2016. Lancet. (2017) 390:1211–59. 10.1016/S0140-6736(17)32154-2 28919117 PMC5605509

[4] Rayner L Hotopf M Petkova H Matcham F Simpson L McCracken L. Depression in patients with chronic pain attending a specialised pain treatment centre: prevalence and impact on health care costs. Pain. (2016) 157:1472–9. 10.1097/j.pain.0000000000000542 26963849 PMC4912238

[5] Smith MT Haythornthwaite JA. How do sleep disturbance and chronic pain inter-relate? Insights from the longitudinal and cognitive-behavioral clinical trials literature. Sleep Med Rev. (2004) 8:119–32. 10.1016/S1087-0792(03)00044-3 15033151

[6] Finan PH Goodin BR Smith MT. The association of sleep and pain: an update and a path forward. J Pain. (2013) 14:1539–52. 10.1016/j.jpain.2013.08.007 24290442 PMC4046588

[7] Fredheim OM Kaasa S Fayers P Saltnes T Jordhoy M Borchgrevink PC. Chronic non-malignant pain patients report as poor health- related quality of life as palliative cancer patients. Acta Anaesthesiol Scand. (2008) 52:143–8. 10.1111/j.1399-6576.2007.01524.x 18005378

[8] Nutt D. Why doctors have a moral imperative to prescribe and support medical cannabis—an essay by David Nutt. BMJ. (2022) 376:n3114. 10.1136/bmj.n3114 35197243

[9] Schlag AK Baldwin DS Barnes M Bazire S Coathup R Curran HV. Medical cannabis in the UK: from principle to practice. J Psychopharm. (2020) 34:931–7. 10.1177/0269881120926677 32522058 PMC7436434

[10] Boychuk DG Goddard G Mauro G Orellana MF. The effectiveness of cannabinoids in the management of chronic nonmalignant neuropathic pain: a systematic review. J Oral Facial Pain Headache. (2015) 29:7–14. 10.11607/ofph.1274 25635955

[11] Finnerup NB Attal N Haroutounian S McNicol E Baron R Dworkin R. Pharmacotherapy for neuropathic pain in adults: a systematic review and meta-analysis. Lancet Neurol. (2015) 14:162–73. 10.1016/S1474-4422(14)70251-0 25575710 PMC4493167

[12] Petzke F Enax-Krumova EK Hauser W. Efficacy, tolerability and safety of cannabinoids for chronic neuropathic pain: a systematic review of randomized controlled studies. Schmerz. (2016) 30:62–88. 10.1007/s00482-015-0089-y 26830780

[13] Whiting PF Wolff RF Deshpande S Di Nisio M Duffy S Hernandez AV. Cannabinoids for medical use: a systematic review and meta-analysis. JAMA. (2015) 313:2456–73. 10.1001/jama.2015.6358 26103030

[14] Häuser W Finn DP Kalso E Krcevsci-Skvarc N Kress H-G Morlion B. European pain federation (EFIC) position paper on appropriate use of cannabis-based medicines and medical cannabis for chronic pain management. Eur J Pain. (2018) 22:1547–64. 10.1002/ejp.1297 30074291

[15] Stockings E Campbell G Hall W Nielson S Zagic D Rahman R. Cannabis and cannabinoids for the treatment of people with chronic noncancer pain conditions: a systematic review and meta-analysis of controlled and observational studies. Pain. (2018) 159:1932–54. 10.1097/j.pain.0000000000001293 29847469

[16] Fisher E Moore RA Fogarty AE Finn D Finnerup N Gilron I. Cannabinoids, cannabis, and cannabis-based medicine for pain management: a systematic review of randomised controlled trials. Pain. (2021) 162:S45–66. 10.1097/j.pain.0000000000001929 32804836

[17] Rice A Arendt-Nielsen L Belton J Blyth F Degenhardt L Di Forti M. International association for the study of pain presidential task force on cannabis and cannabinoid analgesia position statement (IASP). Pain. (2021) 162. 10.1097/j.pain.0000000000002265 33729207

[18] Schlag AK Zafar R Nutt DJ. Medical cannabis and epilepsy in the UK – a qualitative analysis of the carers' perspective: “We're asking for quality of life for our children”. Drug Sci Policy Law. (2021) 7:1–10. 10.1177/20503245211034930

[19] Nutt DJ Phillips LD Barnes MP Brandner B Curran HV Fayaz A. A multicriteria decision analysis comparing pharmacotherapy for chronic neuropathic pain, including cannabinoids and cannabis-based medical products. Cannabis Cannabinoid Res. (2021). 10.1089/can.2020.0129. [Epub ahead of print]. 33998895 PMC9418467

[20] Schlag AK O'Sullivan SE Zafar RR Nutt DJ. Current controversies in medical cannabis: recent developments in human clinical applications and potential therapeutics. Neuropharmacology. (2021) 191:108586. 10.1016/j.neuropharm.2021.108586 33940011

[21] Wiese B Wilson-Poe AR. Emerging evidence for cannabis' role in opioid use disorder. Cannabis Cannabinoid Res. (2018) 3:179–89. 10.1089/can.2018.0022 30221197 PMC6135562

[22] Erridge S Salazar O Kawka M Holvey C Coomber R Usmani A. An initial analysis of the UK medical Cannabis registry: outcome analysis of first 129 patients. Neuropsychopharmacology Rep. (2021) 4193:362–70. 10.1002/npr2.12183 33988306 PMC8411316

[23] Sakal C Lynskey M Schlag AK Nutt DJ. Developing a real-world evidence base for prescribed cannabis in the United Kingdom: preliminary findings from project twenty 21. Psychopharmacology. (2021) 239:1147–55. 10.1007/s00213-021-05855-2 33970291

[24] Takakuwa KM Hergenrather JY Shofer FS Schears RM. The impact of medical cannabis on intermittent and chronic opioid users with back pain: how cannabis diminished prescription opioid usage. Cannabis Cannabinoid Res. (2020) 5:263–70. 10.1089/can.2019.0039 32923663 PMC7480723

[25] Couch D. Left behind: the scale of illegal cannabis use for medicinal intent in the UK. Centre Med Cannabis. (2020). Available online at: https://irp-cdn.multiscreensite.com/51b75a3b/files/uploaded/Centre%20for%20Medicinal%20Cannabis%20%7C%20YouGov%20MC%20Paper%20v3.pdf (accessed May 26, 2022).

[26] Nutt D Bazire S Phillips LD Schlag AK. So near yet so far: why won't the UK prescribe medical cannabis? BMJ Open. (2020) 10:e038687. 10.1136/bmjopen-2020-038687 32958492 PMC7507889

[27] Cleeland C. Brief Pain Inventory. Handbook of Pain Assessment. New York, NY: The Guildford Press (1991). p. 378–82.

[28] Kroenke K Spitzer RL Williams JB. The PHQ-9: validity of a brief depression severity measure. J Gen Intern Med. (2001) 16:606–13. 10.1046/j.1525-1497.2001.016009606.x 11556941 PMC1495268

[29] Herdman M Gudex C Lloyd A Janssen M Kind P Parkin D et al. Development and preliminary testing of the new five- level version of EQ-5D (EQ-5D-5L). Qual Life Res. (2011) 20:1727–36. 10.1007/s11136-011-9903-x 21479777 PMC3220807

[30] Rancans E Trapencieris M Ivanovs R Vrublevska J. Validity of the PHQ-9 and PHQ-2 to screen for depression in nationwide primary care population in Latvia. Annals Gen Psychiatry. (2018) 17:33. 10.1186/s12991-018-0203-5 30083220 PMC6071402

[31] Buysee D Reynolds C Monk T Berman S Kupfer D. The Pittsburgh sleep quality index: a new instrument for psychiatric practice and research. Psychiatry Res. (1989) 28:193–213. 10.1016/0165-1781(89)90047-4 2748771

[32] Serlin RC Mendoza TR Nakamura Y Edwards K Cleeland C. When is cancer pain mild, moderate or severe? Grading pain severity by its interference with function. Pain. (1995) 61:277–84. 10.1016/0304-3959(94)00178-H 7659438

[33] Shi Q Mendoza TR Dueck AC Ma H Zangh J Qian Y. Determination of mild, moderate, and severe pain interference in patients with cancer. Pain. (2017) 158:1108–12. 10.1097/j.pain.0000000000000890 28267060

[34] Devlin NJ Shah KK Feng Y Mulhern B van Hout B. Valuing health-related quality of life: An EQ-5D-5L value set for England. Health Econ. (2018) 27:7–22. 10.1002/hec.3564 28833869 PMC6680214

[35] Börsbo B Peolsson M Gerdle B. The complex interplay between pain intensity, depression, anxiety and catastrophising with respect to quality of life and disability. Disabil Rehabil. (2009) 31:1605–13. 10.1080/09638280903110079 19848559

[36] Aviram J Lewitus GM Vysotski Y Yellin B Berman P Shapira A. Prolonged medical cannabis treatment is associated with quality of life improvement and reduction of analgesic medication consumption in chronic pain patients. Front Pharmacol. (2021) 12:613805. 10.3389/fphar.2021.613805 34093173 PMC8172141

[37] Schlienz NJ Scalsky R Martin EL Jackson H Munson J Strickland J. A cross-sectional and prospective comparison of medicinal cannabis users and controls on self-reported health. Cannabis Cannabinoid Res. (2021) 6:548–58. 10.1089/can.2019.0096 33998852 PMC8713273

[38] Lucas P Baron EP Jikomes N. Medical cannabis patterns of use and substitution for opioids & other pharmaceutical drugs, alcohol, tobacco, and illicit substances; results from a cross- sectional survey of authorized patients. Harm Reduct J. (2019) 16:9. 10.1186/s12954-019-0278-6 30691503 PMC6350348

[39] Hsu G Kovács B. Association between county level cannabis dispensary counts and opioid related mortality rates in the United States: panel data study. BMJ. (2021) 372:m4957. 10.1136/bmj.m4957 33504472 PMC7838036

[40] Corroon Jr JM Mischley LK Sexton M. Cannabis as a substitute for prescription drugs - a cross- sectional study. J Pain Res. (2017) 10:989–98. 10.2147/JPR.S134330 28496355 PMC5422566

